# A Reliable Hybrid Adsorbent for Efficient Radioactive Cesium Accumulation from Contaminated Wastewater

**DOI:** 10.1038/srep19937

**Published:** 2016-01-28

**Authors:** Md. Rabiul Awual, Tsuyoshi Yaita, Yuji Miyazaki, Daiju Matsumura, Hideaki Shiwaku, Tomitsugu Taguchi

**Affiliations:** 1 Actinide Chemistry Group, Japan Atomic Energy Agency (SPring–8), Hyogo 679–5148, Japan; 2 Environment and Materials Dynamics Research Group, Japan Atomic Energy Agency (SPring–8), Hyogo 679–5148, Japan; 3 Laser Processing Research Group, Quantum Beam Science Center, Japan Atomic Energy Agency (JAEA), Tokai–mura, Ibaraki–ken 319–1195, Japan.

## Abstract

Cesium (Cs) removal from nuclear liquid wastewater has become an emerging issue for safeguarding public health after the accident at the Fukushima Daiichi Nuclear Power Plant. A novel macrocyclic ligand of *o*-benzo-*p*-xylyl-22-crown-6-ether (OBPX22C6) was developed and successfully immobilized onto mesoporous silica for the preparation of hybrid adsorbent. The benzene ring π electron is the part of crown ether of OBPX22C6 for easy orientation of the macrocyclic compound for making the π electron donation with Cs complexation. The potential and feasibility of the hybrid adsorbent as being Cs selective was evaluated in terms of sensitivity, selectivity and reusability. The results clarified that the Cs removal process was rapid and reached saturation within a short time. Considering the effect of competitive ions, sodium (Na) did not markedly affect the Cs adsorption whereas potassium (K) was slightly affected due to the similar ionic radii. However, the oxygen in long ethylene glycol chain in OBPX22C6 was expected to show strong coordination, including Cs-π interaction with Cs even in the presence of the high amount of K and Na. Due to its high selectivity and reusability, significant volume reduction is expected as this promising hybrid adsorbent is used for Cs removal in Fukushima wastewater.

Large amounts of radioactive elements were released after the Fukushima Daiichi nuclear power plant disaster in 2011. Among those radioisotopes, cesium (Cs) and especially ^137^Cs has spread out extensively both in soil particles and surface waters^1^. The Cs has posed serious environmental public-health threats due to its high transportability via the atmosphere, long half-life (30.4 years), and high solubility and movability within aqueous media. Also the Cs has large bioavailability, which is very similar to potassium (K) ions and Cs can readily be assimilated by terrestrial and aquatic organisms^2^. Therefore, Cs can easily enter the human body and remain for long periods, potentially irradiating living tissue. Then several diseases are reported due to the adverse effect of radionuclides.^3^,^4^,^5^. Thus, the removal of Cs in polluted environments needs to be resolved with urgency. However, in aqueous media, Cs is present as free ions and its speciation is unaffected even after changes of solution pH or redox conditions^6^. As a result, the selective separation of low concentration levels of radioactive Cs has attracted the attention of many researchers and scientist due to the co-existence of high concentrations of K and sodium (Na) ions. Therefore, adsorbent materials that can selectively and efficiently remove this radionuclide from processed and nuclear wastewaters before being discarding into bodies of water is important for minimizing water pollution caused by radionuclides.

Several methods have been proposed for the separation and removal of ^137^Cs from nuclear liquid waste such as evaporation, co-precipitation, solvent extraction, chemical treatment, ion-exchange, micro-filtration and membrane processes^7^,^8^,^9^,^10^,^11^,^12^. In the solvent extraction method, several crown ethers are frequently investigated due to their strong extraction and complexing ability to Cs. Also the cavity of its ligand matches well with the ionic radius of Cs and separation factors are observed^10^. However, the traditional solvent extraction process may have disadvantages such as radiolysis degradation, use of large-scale equipment and generation of abundant organic waste. Moreover, incomplete removal, high cost and considerable disposal of toxic waste resulting from such processes have significantly hindered the application of these methods. Various ion-exchange resins such as hollow Prussian blue, zeolite, ammonium molybdophosphate-polyacrylonitrile (AMP-PAN), potassium-nickel hexacyanoferrate-polyacrylonitrile (KNiFC-PAN), metal hexacyanoferrate and polyphenol rich have also been investigated for removing Cs from liquid wastewater^13^,^14^,^15^,^16^,^17^,^18^,^19^,^20^,^21^,^22^,^23^. However; highly selective materials are always welcome to remove the Cs from the liquid wastewater. Furthermore, it has been noted that not only the Cs ion but also other metal ions can be exchanged, which leads to a waste of adsorption capacity and inability to remove Cs under high ionic strength conditions such as sea water. Extensive research has been carried out for selective Cs adsorption on various materials^24^,^25^. Among these materials, organic-inorganic materials have received more attention due to their structural and economic advantages, such as large quantity, low cost, physical stability and high adsorption capacity^26^,^27^,^28^.

A coordination mechanism based on cesium complex with high coordination numbers is desirable for selective Cs separation. The macrocyclic crown ethers are the best-suited organic material for their application as host molecules for efficient Cs separation from the waste sample even from acidic and alkaline nuclear waste solutions^29^,^30^. The crown ethers ligands have shown high selectivity towards Cs as compared to Na ions due to cation-π electron interactions^31^,^32^. Novel cesium selective ligands with crown families have been introduced^31^,^33^,^34^. These compounds have demonstrated significant complexation ability between the Cs-π interactions of the aromatic rings^35^,^36^,^37^. This led us to introduce new cesium-selective crown ethers. Then a new class macrocyclic ligand of *o*-benzo-*p*-xylyl-22-crown-6-ether (OBPX22C6) was developed (Fig. 1). The OBPX22C6 has the ability to identify the Cs ion size based on cation-π interaction. The π electron of the benzene ring in OBPX22C6 has high tendency to interact with d-f hybrid orbital electron of Cs. Moreover, the d-f hybrid orbital is absent in Na and K ions. Therefore, the Cs can easily interact with the π electron of the benzene ring in OBPX22C6 including the oxygen donation of the long ethylene glycol and Cs can selectively be captured from wastewater even in the presence of the high amount of K and Na ions.

There is a growing interest in the application of nanomaterials as effective adsorbents for the removal of pollutants due to the different types of physicochemical properties. In nanomaterials, most of the atoms on the surface are unsaturated and can bind to other atoms by hydrogen bonding and heteroatoms. Nanomaterials have also shown high performance for the removal of contaminants because of their high surface area, large pore volume, high pore sizes and absence of internal diffusion resistance^38^,^39^. Therefore, when nanomaterials are immobilized with functional organic compounds, it creates a new powerful class of hybrid nanomaterials that can be used for the purpose of the fast and potential remediation process^39^,^40^,^41^. To improve the rate and selectivity of Cs adsorption at extremely low concentrations onto adsorbents, adsorbents with specific functional groups containing organic ligand are preferable with extreme selectivity based on the inorganic material supported hybrid adsorbent^27^.

In the current research, an approach is made for high selectivity with a further increase the Cs adsorption efficiency. A new type of OBPX22C6 was embedded onto mesoporous material. The organic-inorganic base hybrid adsorbent possesses high functionality and thus Cs can be removed easily even in the presence of extremely similar co-existing alkaline ions. With a view to bring alternative methods, the organic-inorganic based adsorbents are suitable materials for selective Cs separation from the nuclear liquid waste^26^,^34^,^42^,^43^,^44^. The major advantages of the hybrid adsorbent are simultaneous separation and regeneration, less waste production, easy scaling-up and low power consumption^27^,^45^. A series of experiments was carried out to assess the utility of this prepared hybrid adsorbent for the removal of Cs from aqueous solutions. The present work investigated the hybrid adsorbent’s performance in the removal of Cs under different experimental conditions such as contact time, initial solution pH, adsorbent dosage, initial Cs concentration, adsorption equilibrium, competitive cations and reuse in detail.

## Results and discussion

### Novelty of the hybrid adsorbent

After the accident in Fukushima power plant, the reactor was being cooled using sea water. However, the sea water contained a huge amount of Na and K. Moreover, Cs is also one of the alkali metal ions based on the periodic table. In the mesoporous silica, the abundant hydroxyl groups were bonded with OBPX22C6 by heteroatoms. However, the all hydroxyl groups were unable to coordinate with ligand molecule and the free hydroxyl group was also coordinated with Cs. On the other hand, in OBPX22C6, the benzene ring π electron is the part of crown ether for parasailing in place by concatenating while the oxygen in methylene ether in the non-shared electron pairs in easy orientation of the macrocyclic compound for making π electron donation with Cs complexation as depicted in Fig. 2. Also the oxygen in long ethylene glycol chain emphasizes the strong coordination with Cs even in the presence of a high amount of K and or Na ions. The interaction of Cs-π is one of the oxygen atoms leaning toward the benzene ring. However, the cation-π interactions are observed in several adducts of the alkali metal cation with neutral aromatic molecules, in particular, when the benzene ring carries an electron rich substituent. As with other noncovalent interactions involving aromatic systems, the Cs-π interaction includes a substantial electrostatic component^46^. Therefore, it is postulated that the developed hybrid adsorbent has significant functionality for selective Cs separation from the multi-mixture solutions based on the stable complexation mechanism and mesoporous silica was kept the ligand functionality to act as hybrid adsorbent based on ligand and mesoporous silica binding ability.

### Mesoporous silica and hybrid adsorbent

The N_2_ adsorption-desorption isotherms measurement was carried out to understand the insight into the porosity, pore volumes and the specific surface area of the mesoporous silica. The textural parameters of mesoporous silica are listed in Fig. 3 with isotherms behavior. The mesoporous silica possesses a total surface area and specific pore volume. The high surface area and pore volume of mesoporous silica observed is consistent with the previously reported literature^47^,^48^,^49^. After immobilization of OBPX22C6, both the specific surface area and pore volume decreased as expected (Fig. 3 (inset)) where macrocyclic organic compound was embedded into the mesoporous silica. All these changes may be a consequence and the hybrid adsorbent exhibited appreciated porous structure for Cs adsorption from aqueous solution. Both mesoporous silica and hybrid adsorbent present IV type isotherms with H_2_ hysteresis loop according to the international union of pure and applied chemistry (IUPAC) classification, which is characteristic of mesoporous materials with interconnected pore geometry and a high energy of adsorption^48^. In addition, the larger surface area of hybrid adsorbent provides more available adsorption sites for Cs ions. The hybrid adsorbent can take up Cs ions with inner sphere and outer sphere complexes with high flexibility.

Figure 4(A,B) shows the images of mesoporous silica using scanning electron microscopy. The prepared mesoporous silica particles were uniformly spherical with a mean diameter according to the scanning electron microscopy (SEM) images, which vividly showed the ordered mesoporous platform. The transmission electron microscopy (TEM) images also (Fig. 4(C,D)) showed ordered channels morphology with hexagonal pore structure. These results suggested that mesoporous silica was nucleated homogeneously and OBPX22C6 ligand could be embedded on the surface successfully. Figure 4(E,F) shows the STEM images of the hybrid adsorbent. After ligand immobilization, the hexagonal porous structure was sufficient leading to easy migration of into the interior of the hybrid adsorbent composites, which could enhance the adsorption capacity of the Cs ions.

### Effect of solution acidity

Solution acidity is an important parameter in the removal of metal ions from water solution due to the effect of metal speciation and ionization of the functional ligand. However, the Cs has existed as Cs(I) at all pH ranges^50^. From Fig. 5(A), the Cs removal efficiency was decreased dramatically in the acidic pH region. However, the Cs adsorption efficiency is obviously positive by the hybrid adsorbent and the adsorption efficiency increased with increasing the initial pH of the solution. This phenomenon is probably attributed to the competitive behavior between H_3_O^+^ and Cs during the adsorption operation under acidic area^28^,^51^. The data clarified that the Cs adsorption efficiency was high at the neutral pH region due to the competition of H_3_O^+^ decreases. Moreover, the alkaline media was not considered due to the pH solution adjustment using sodium hydroxide (NaOH) solution, where Na was competitive ions on the Cs adsorption in this study. In addition, the maximum Cs removal was observed at pH 7.0, thus all experiments were performed at pH 7.0 under this optimum condition.

### Effect of contact time

Adsorption rate is one of the most important characteristics of the adsorbent for representing the adsorption efficiency. In most cases, the evaluation adsorption rate is based on determining equilibrium time for the metal ions adsorption. However, different adsorbents exhibited their own equilibrium times because of the different physical and chemical characteristics which consisted of surface area, pore size and surface charge based on the adsorption mechanism. In the present study, the effect of contact time on Cs adsorption by the hybrid adsorbent was evaluated when the initial concentration was constant at 0.038 mM. The Cs adsorption was increased with time and thus remains constant as judged from Fig. 5(B). Also, the Cs adsorption increased with time and gradually reached the equilibrium value. Further increase in contact time had a negligible effect on the Cs adsorption by the adsorbent. The data also confirmed that the hybrid adsorbent exhibited rapid Cs adsorption with an equilibrium time of approximately 30 min. This data was significant compared to other forms adsorbent materials such as clay minerals of aluminum-pillared montmorillonite, zeolite, pristine and copper hexacyanoferrate polyacrylonitrile^8^,^52^. This data also clarified that the equilibrium time was found to be independent of the initial concentration. Therefore, the contact time was fixed at 3 h for the rest of the batch experiments to make sure that the equilibrium was reached when the initial concentration was high in the evaluation of maximum adsorption capacity by the adsorbent.

### Effect of competing ions

Ion selectivity is an important parameter for the adsorbent because the adsorption operation will be affected in the presence of co-existing metal ions considering real liquid waste-water treatment. Considering the ionic radii of Cs (1.69 Å), K (1.33 Å) and Na (0.95 Å), the K will be highly competing during Cs adsorption by the adsorbent. In natural waters (sea and surface waters), Na and K are abundantly present and this has great negative effects on Cs adsorption by the hybrid adsorbent. Moreover, the Cs concentration is much lower than the Na or K even in the radioactive contaminated wastewaters in Fukushima wastes samples. Figure 6 shows the effect of Na and K on Cs adsorption efficiency by the hybrid adsorbent. It is also noted that the Cs concentration was fixed at 0.0075 mM while the K concentration was varied from 0.025 mM to 7.69 mM and the Na concentration was varied from 0.043 mM to 13.04 mM. The data clarified that by increasing the K concentration, the Cs adsorption decreased as judged from Fig. 6(A). Even in the presence of 7.69 mM K (1,025 fold), the Cs removal efficiency was more than 58%. The presence of K is strongly affected the Cs adsorption as reported by the other investigations^53^. The hydration energy and similar ionic radii between Cs and K result in strong competition against Cs on the adsorbent surface^54^. The Na also affects the Cs adsorption by the adsorbent to a certain concentration (13.04 mM while the Cs concentration was 0.0075 mM) due to the high content in water samples. As we expected, Na is less competitive rather than K. Therefore, Cs removal efficiency was more than 72% when the initial Cs concentration was 0.0075 mM even in the presence of 13.04 mM Na (1,739 fold) (Fig. 6(B)). Under the optimum conditions, the Cs adsorption on hybrid adsorbent is much higher than on zeolites. These results confirmed that the novel hybrid adsorbent has high selectivity to the Cs ions compared with the reported literature due to the specific Cs-π interaction as already discussed in the preceding section. Therefore, the newly developed hybrid adsorbent will have a major impact on the removal of radioactive Cs from the nuclear liquid waste in Fukushima, Japan. Based on the data presentation, the developed hybrid adsorbent is able to decontaminate the real Cs contaminated nuclear waste sample as expected in our earlier reported works^26^,^27^.

### Effect of initial concentration and adsorption capacity

Equilibrium adsorption studies were performed at different Cs concentration ranging from 0.015 to 1.13 mM. Figure 7(A) shows that the Cs adsorption increased with the increase of the initial Cs concentration. A high initial concentration is affected by the driving force caused by the concentration gradient and mass transfer effects and then the Cs adsorption values increase. The data also emphasized that the equilibrium Cs adsorbed on the hybrid adsorbent with increasing initial concentrations. This means that the Cs adsorption obeys the Langmuir adsorption equation where adsorption sites are uniformly energetic, and the coverage is a monolayer without tangential interactions between adsorbed molecules^55^. The equilibrium isotherm of Cs on hybrid adsorbent was modeled by Langmuir models. The following linear form of Langmuir adsorption isotherms model was fitted with the experimental data.


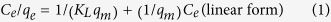


where *q*_*e*_ (mg/g) is the adsorption capacity at equilibrium, *Ce* (mg/L) is the equilibrium Cs ions concentration, and *q*_*m*_ (mg/g) and *K*_*L*_ (L/mg) are the Langmuir constants related to the maximum adsorption capacity and energy of adsorption, respectively. The values of *q*_*m*_ and *K*_*L*_ were calculated from the slope and intercept of the linear plot of Ce/qe versus Ce. The data analyses based on the correlation coefficients (*R*^*2*^ = 0.986) were fitted well with the Langmuir model as judged from Fig. 7(A) (inset). This means that the adsorption process is a monolayer adsorption^56^. The maximum adsorption value was calculated to be 86.28 mg/g. The radioactive concentration is low in the real liquid waste sample, and the adsorption capacity by the hybrid adsorbent is promising and meaningful for efficient Cs removal from waste solutions. Also the high adsorption capacity indicated that the hybrid adsorbent exhibits the high functionality to remove Cs in terms of high adsorption thus making the adsorbent a promising candidate for potential applications to *in situ* nuclear liquid waste treatment in Fukushima, Japan.

Table 1 shows the comparison of maximum adsorption capacity of the hybrid adsorbent with other forms of adsorbents. The comparison provides some indication as to the use of the potential adsorbent in real radioactive liquid waste treatment. However, the maximum adsorption capacity also depends on the Cs concentration, solution acidity, adsorbent functionality and the nature of the foreign ions. The OBPX22C6 immobilized inorganic-organic based hybrid adsorbent exhibits comparatively higher Cs adsorption capacity than the different forms of adsorbents. Several types of adsorbents such as hollow Prussian blue, Zeolite A, AMP-PAN and KNiFC-PAN are also exhibit high adsorption capacity^22^,^57^,^58^. Based on the high adsorption capacity, the present hybrid adsorbent is promising for environmental remediation.

### Elution, regeneration and reuses

Elution operation indicates the recovery of adsorbed metal ions and simultaneously regenerates the adsorbent for understanding the nature of the adsorption process. Figure 7(B) shows that Cs loaded on the hybrid adsorbent can be eluted with 0.25 M HCl solution. The elution of Cs by acidic solution clarifies that the Cs is adsorbed by chemisorptions method. The data also clarified that 97% Cs was recovered in the second cycle; a reduction of 1.6% compared with the first cycle of the adsorption efficiency. The adsorption efficiency decreased during the six adsorption-elution cycles where the elution efficiency reduction was less than 10%. After six cycles, the Cs adsorption efficiency of hybrid adsorbent remains at 91%. Therefore, the hybrid adsorbent can be reused many cycles without much loss of its adsorption capacity. The adsorption-elution process of Cs is schematically shown in Fig. 8. The major advantage of hybrid adsorbent is their retention of functionality in terms of selection and removal activity after multiple regeneration cycles.

## Conclusions

The novel crown ether based hybrid adsorbent was considered as effective adsorbent materials for efficient cesium (Cs) separation in terms of selectivity and reusability. The adsorbent exhibited larger surface area and large pore sizes for easy incorporation Cs with specific functionality of organic compound. The solution pH exhibited an important parameter of the Cs adsorption on hybrid adsorbent with an optimum pH at 7.0. The adsorption isotherms were well fitted by Langmuir isotherm model and the calculated maximum adsorption capacity was 86.28 mg/g. The presence of K and Na slightly inhibited the Cs adsorption by the hybrid adsorbent due to the similar ionic radii. However, more than 60% of the Cs could be adsorbed even in the presence of high concentrations of K (7.69 mM) and Na (13.04 mM). The high selectivity was estimated by the Cs-π interaction of the OBPX22C6 benzene ring π electron on the hybrid adsorbent surface. The high adsorption capacity, rapid adsorption process, high selectivity and high reusability of the hybrid adsorbent imply that this adsorbent could be used as an effective adsorbent for Cs removal from radioactive aqueous waste in Fukushima.

## Experimental

All materials and chemicals were of analytical grade and used as purchased without further purification. Tetramethylorthosilicate (TMOS) and the triblock copolymers of poly(ethylene oxide–b–propylene oxide–b–ethylene oxide) designated as F108 (EO_141_PO_44_EO_141_) were obtained from Sigma–Aldrich Company Ltd. USA. Cesium chloride (CsCl), *p*-xylene-α,α‘-diol, and metal salts for the source of metal ions were purchased from Wako Pure Chemicals, Osaka, Japan. The bis(2-chloroethyl)ether and catechol were obtained from Tokyo Chemical Industry Co., Ltd. (TCI), Japan. Ultra–pure water prepared with a Millipore Elix Advant 3 was used throughout this work.

### Synthesis of o-benzo-p-xylyl-22-crown-6-ether (OBPX22C6)

**Figure 1:**
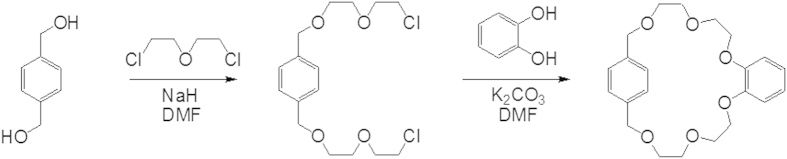
Synthetic route for the preparation of macrocyclic *o*-benzo-*p*-xylyl-22-crown-6-ether (OBPX22C6) ligand.

**Figure 2:**
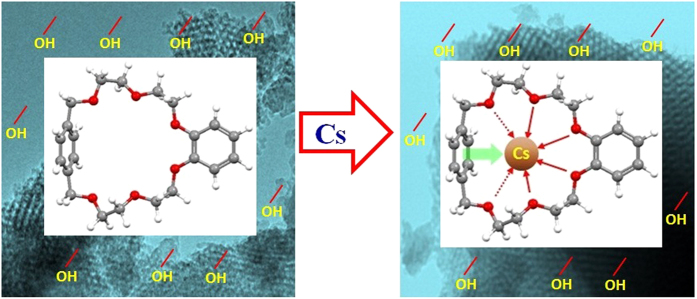
Representative of Cs complexation with hybrid ligand consisting of OBPX22C6 using Cs-π interaction for selective Cs removal in the presence of high amount of alkaline potassium (K) and or sodium (Na) ions.

**Figure 3:**
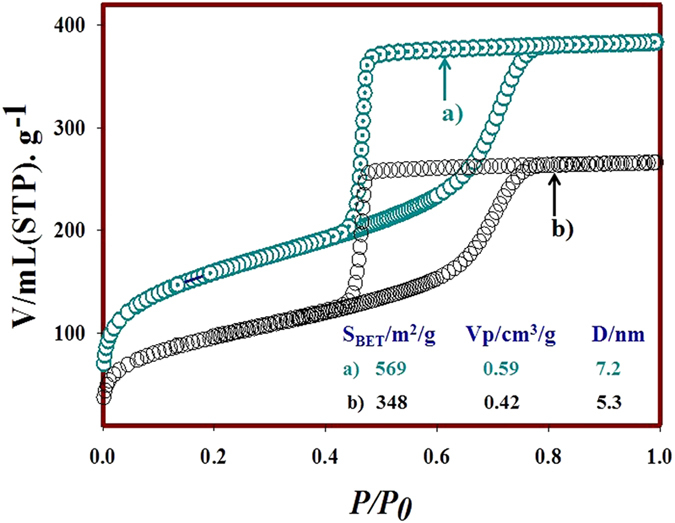
Nitrogen adsorption-desorption isotherms at 77 K of (a) mesoporous silica monolith and (b) hybrid adsorbent after successful immobilization of OBPX22C6 macrocyclic ligand, respectively. The insert lists in (a,b) are the surface area (*S*_*BET*_), pore volume (*Vp*), and the pore size (D) of the mesoporous silica and hybrid adsorbent, respectively.

**Figure 4:**
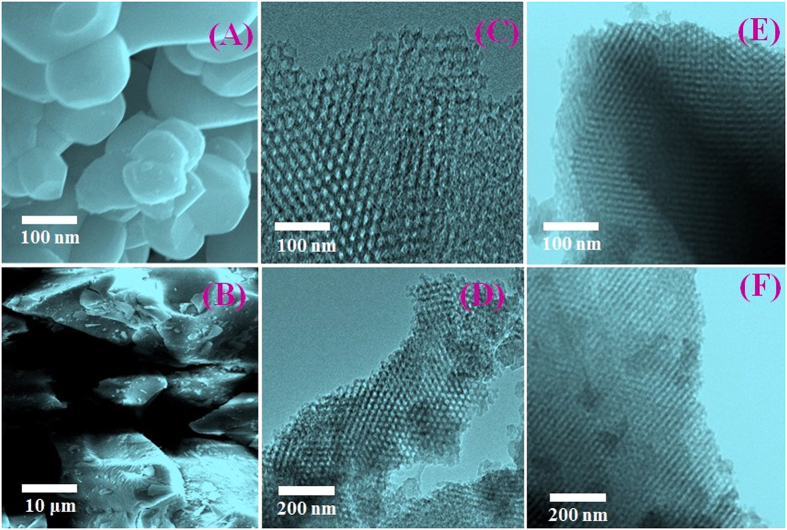
Representative of SEM(**A,B**) and TEM (**C,D**) images of parallel hexagonal pore channel systems uniformly shaped mesoporous silica; STEM images of hybrid adsorbent also representing the well-ordered mesoporous structure for high order Cs capturing from wastewater samples.

**Figure 5:**
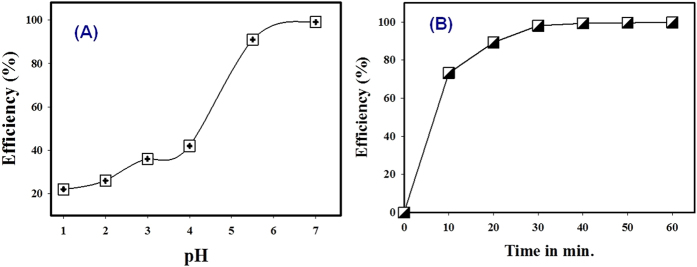
Effect of solution acidity pH on cesium adsorption by the hybrid adsorbent (**A**) and Variation of Cs adsorption on the adsorbent as a function of contact time (**B**) where the initial Cs concentration: 0.03 mM and 0.038 mM for pH effect and contact time evaluation, respectively; amount of adsorbent: 10 mg and solution volume: 10 mL.

**Figure 6:**
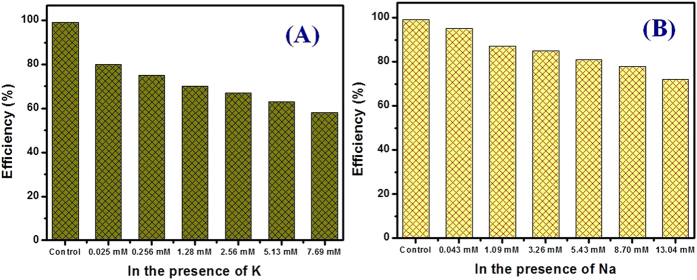
Ion selectivity effect of K (A) and Na (B) on the Cs removal efficiency by the hybrid adsorbent. At optimum conditions where the initial Cs concentration fixed at 0.0075 mM, amount of adsorbent: 10 mg; solution volume: 10 mL; K concentration: 0.025 mM to 7.69 mM; Na concentration: 0.043 mM to 13.04 mM.

**Figure 7:**
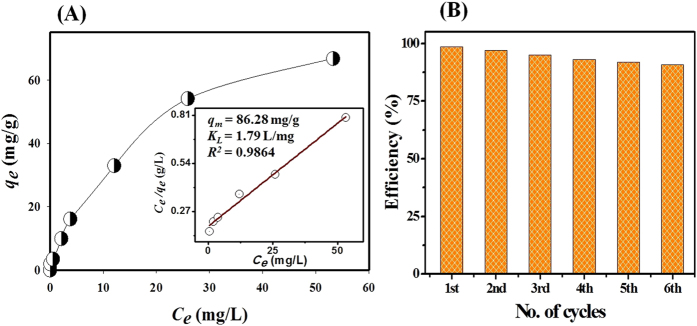
(**A**) Effect of initial concentration of the cesium adsorption on the hybrid adsorbent and fitting of Langmuir isotherm with cesium adsorption where the initial Cs concentration was varied from 0.015 mM to 1.13 mM; solution pH 7.0; adsorbent amount 10 mg; solution volume 10 mL, shaking time for 3 h and (**B**) the elution operation using 0.25 M HCl solution and recycling the hybrid adsorbent during six adsorption-elution-regeneration cycles.

**Figure 8:**
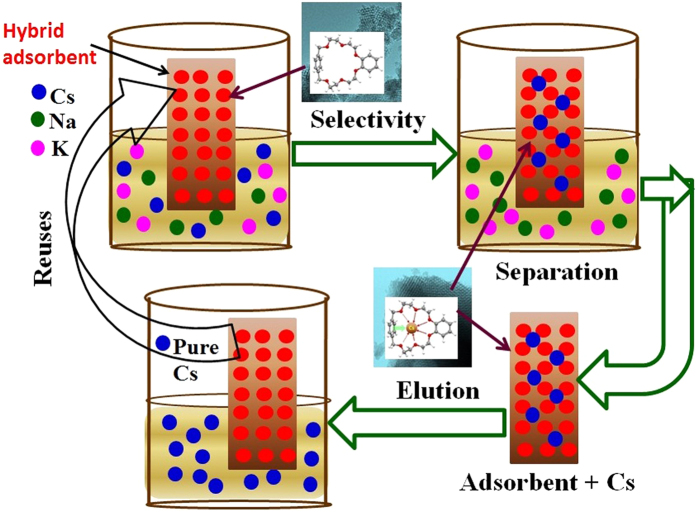
A schematic design of the construction of ligand based hybrid adsorbent for Cs removal and recyclable of the adsorbent from the stand point of long time uses without significant loss of the performances.

The preparation steps for *o*-benzo-*p*-xylyl-22-crown-6-ether (OBPX22C6) are shown Fig. 1. In a round bottom flask, NaH (1.01 g, 42 mmol) was mixed in DMF (10 mL) solvent. Then *p*-xylene-α,α‘-diol (2.76 g, 20 mmol) in DMF (50 mL) solution was added slowly, and the mixture was stirred at room temperature for 1 h. After that bis(2-chloroethyl)ether (28.6 g, 200 mmol) was added into the mixture and stirred at room temperature for 24 h. Then the mixture solution was cooled at 0C and methanol (10 mL) was added in this stage. The organic solution was evaporated using rotary evaporation and the residue compound was dissolved in dichloromethane and water. The crude product was purified by column chromatography using hexane/ethyl acetate as ration of 2:1 and separated the desired product of *p*-xylene-α,α‘-di(ethylene glycol mono-2-chloroethylether). In a three neck flask K_2_CO_3_ (4.63 g, 33.5 mmol), NaI (402 mg, 2.7 mmol) in acetonitrile (218 mL) were mixed and the mixture was refluxed. A solution of *p*-xylene-α,α‘-di(ethylene glycol mono-2-chloroethylether) (4.72 g, 13.4 mmol) and catechol (1.48 g, 13.4 mmol) in acetonitrile (50 mL) was added slowly and then the mixture was refluxed for 4 days. After cooling at room temperature, the mixture was filtrated and inorganic salt was separated. In addition, the organic solution was also evaporated. The crude product was purified by column chromatography on silica gel using hexane/ethyl acetate and OBPX22C6 was separated accordingly. The product was characterized by ^1^H NMR spectroscopy. The ^1^H NMR (400 MHz, CDCl_3_): δ 7.36 (s, 4H), 6.89 (br-s, 4H), 4.57 (s, 4H), 4.10 (t, *J* = 5.2 Hz, 4H), 3.64 (t, *J* = 4.6 Hz, 4H), 3.63 (t, *J* = 5.2 Hz, 4H), 3.55 (t, *J* = 4.6 Hz, 4H).

### Instruments and analyses

The NMR spectra were obtained on a Varian NMR System 400 MHz Spectrometer. The SEM analysis was performed on Hitachi S-4300 operated at 16 keV. TEM images was obtained by using a JEOL (JEM-2100F) and operated at the accelerating voltage of the electron beam 200 kV. The N_2_ adsorption-desorption isotherms were measured using the 3Flex analyzer (Micromeritics, USA) at 77 K. The metal ion concentrations were measured by atomic absorption spectrophotometer (AAS, Hitachi, Z-2300).

### Preparation of mesoporous material and hybrid adsorbent

Mesoporous silica was prepared by using direct templating method of lyotropic liquid crystalline phase where F108 surfactant was used as the scaffolding template. The mesoporous silica was synthesized following the reported methods with slight modification^59^,^60^. Under typical conditions, the composition mass ratio of F108:TMOS:HCl/H_2_O was 1.2:2:1 respectively. Homogeneous sol-gel synthesis was achieved by mixing of F108/TMOS in a round balloon flask and then shaking at 60 °C until homogeneous. The hydrolysis and condensation occurred after addition of HCl (at pH = 1.3) to this homogeneous solution. Then the methanol produced from the TMOS hydrolysis was removed by a rotary evaporator at 45 °C. The organic moieties were removed by calcination at 500 °C for 6 h under normal atmospheric condition. Then the material was ground gently and ready to use for fabrication hybrid adsorbent with OBPX22C6 ligand immobilization.

The hybrid adsorbent was prepared by direct immobilization approach. The OBPX22C6 (50 mg) was added to the *N, N*–dimethylformamide (DMF) solution and then 1.0 g of mesoporous silica was added to the solution. The immobilization procedure was performed under vacuum at 55 °C until OBPX22C6 saturation was achieved. The DMF was removed by a rotary evaporator at 80 °C and the hybrid adsorbent was washed in warm water to check the leaching and elution of OBPX22C6 from the mesoporous silica. The material was then dried at 45 °C for 6 h and ground to fine powder for Cs ions removal experiments to justify the several parameters under optimal conditions.

### Cesium removal

The pH solution was adjusted by adding HCl as required. To determine the adsorption efficiency, a fixed amount of hybrid adsorbent was added Cs ion containing 10 mL solutions and shook for 50 min (except for the initial concentration effect) at room temperature. In cases of pH effect evaluation, the initial Cs concentration was 0.03 mM, while the contact time effect, the Cs concentration was 0.038 mM. After shaking the mixture, the conjugate adsorbent was separated and supernatant concentration was analyzed. The Cs adsorption was determined based on the following equations:


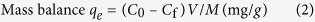


and Cs sorption efficiency


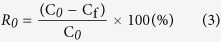


where *V* is the volume of the aqueous solution (L), and *M* is the weight of the hybrid adsorbent (g), C_*0*_ and C_*f*_ are the initial and supernatant Cs concentrations in solution, respectively.

To determine the regeneration and reusability of the hybrid adsorbent, elution experiments were performed. Firstly, a 10 mL of 3 mM Cs containing solution was adsorbed by the 40 mg hybrid adsorbent and then the elution experiments were carried out with 0.25 M HCl acid. The hybrid adsorbent containing Cs was washed with deionized water and transferred into a 50 mL beaker. Then 5.0 mL of the eluting agent was added, and the mixture was stirred for 15 min. The concentration of Cs ions released from the hybrid adsorbent into aqueous phase was analyzed by AAS. It is also noted that all experiments in this study were duplicated to assure the consistency and reproducibility of the results.

## Additional Information

**How to cite this article**: Awual, M. R. *et al*. A Reliable Hybrid Adsorbent for Efficient Radioactive Cesium Accumulation from Contaminated Wastewater. *Sci. Rep.* **6**, 19937; doi: 10.1038/srep19937 (2016).

## Figures and Tables

**Table 1:** Comparison of Cs adsorption capacities with different forms of adsorbents.

**Used adsorbent materials**	**Capacity (mg/g)**	**References**
Walnut shell	3.99	[4]
Hollow Prussian Blue	262.0	[14]
AMP-PAN	64.0	[19]
KNiFC-PAN	256.0	[20]
Persimmon waste	101.08	[22]
Conjugate adsorbent	50.23	[26]
Conjugate adsorbent	65.06	[27]
Silica-based calix[4] arene-R14	20.75	[42]
Nano manganese oxide	65.00	[57]
Zeolite A	222.11	[58]
Hybrid adsorbent	86.28	This study
